# Synergistic and Antagonistic Effects of Insecticides and Heavy Metals on the Bird Cherry–Oat Aphid, *Rhopalosiphum padi* (L.), Under Laboratory Conditions

**DOI:** 10.3390/toxics14070559

**Published:** 2026-06-26

**Authors:** Mohammed A. A. Saad, Verónica Andrade-Yucailla, Salma A. Abdeen, Hala H. Gomah, Hosam A. EzzEl-Din, Mohamed Ahmed Ibrahim Ahmed, Ahmed M. M. Ahmed

**Affiliations:** 1 Department of Plant Protection, Faculty of Agriculture, Assiut University, Assiut 71526, Egypt; mohamed.abdelbasset@agr.aun.edu.eg (M.A.A.S.); salma@agr.aun.edu.eg (S.A.A.); hossameldeen.ahmed@agr.aun.edu.eg (H.A.E.-D.); drmaiaf2000@aun.edu.eg (M.A.I.A.); 2 Centro de Investigaciones Agropecuarias, Facultad de Ciencias Agrarias, Universidad Estatal Península de Santa Elena, La Libertad 204102, Santa Elena, Ecuador; 3 Department of Soil and Water, Faculty of Agriculture, Assiut University, Assiut 71526, Egypt; hala.hasanian@agr.aun.edu.eg

**Keywords:** aphid, heavy metals, insecticides, toxicity

## Abstract

Co-exposure to insecticides and heavy metals alters the way the chemicals affect the aphid. This interaction can induce synergism through enzymatic inhibition of the insect, increasing mortality, or antagonism through competition for cellular receptors. The aim of this study was to determine the synergistic or antagonistic effect of four insecticides and five heavy metals on the median lethal concentrations (LC_50_ and LC_90_) and their toxic efficiency between 24 and 48 h post-exposure to the bird cherry–oat aphid under laboratory conditions. The four pesticides were acetamiprid, imidacloprid, emamectin benzoate, and thiamethoxam. The five heavy metals were Cr, Ni, Pb, Cd, and Cu. The results revealed LC_50_ and slope values for all four tested insecticides against the aphid. After 24 and 48 h from treatment, acetamiprid was the most toxic compound according to LC_50_ values, followed by emamectin benzoate, thiamethoxam, while imidacloprid was the least toxic one. The results indicated that thiamethoxam was synergized by Cr, Cu, Cd, Ni, and Pb by 18.31, 5.36, 74.12, 4.58, and 225.46-fold, respectively, as compared with LC_50_ values of thiamethoxam alone. This finding indicated that Pb was the most effective heavy metal in synergizing thiamethoxam. The results indicated that the toxicity of imidacloprid increased by adding each of the tested heavy metals except for Cu as compared with LC_50_ of imidacloprid alone. Results showed that LC_50_ value of each insecticide decreased with Cd as compared with LC_50_ value of the insecticide alone at both 24 h and 48 h. Under the conditions of this study, it can be concluded that the Cd acted as a synergist for all tested insecticides.

## 1. Introduction

Wheat is one of the most important food crops for human and animal feeding. The Egyptian cultivated area of the wheat crop is approximately 1,369,038 ha, producing about 9 million tons [1]. Globally, wheat ranks among the top three cereal crops, with world production exceeding 780 million tons annually, making it a cornerstone of food security across more than 100 countries [2]. Wheat crops are attacked by many insect pests that cause great damage and reduce the yield. There are 29 aphid species that were recorded on several wheat cultivars [3]. Specially, the bird cherry oat aphid: *Rhopalosiphum padi* (L.) is the most abundant one recorded on different wheat cultivars and considered a serious insect key pest attacking wheat in Upper Egypt [4]. The direct loss of damage occurred by aphids on wheat plantation up to 35–40% with direct reduction in total numbers of wheat heads, grains/head, and grains net weight [5,6]. The indirect loss occurs as a second infestation by virus transmission while feeding by inserting their stylet on plant cells and tissues, triggering reactions between infested and healthy plants followed by the growth of fungi and molds affecting photosynthesis [7]. Solely, *R. padi* species are able to cause damage up to 600 kg/ha in yield of wheat crops [8] and transmit barley/cereal yellow dwarf virus (B/CYDV) causing yellow dwarf disease (YDD) [9].

Insecticides are a widely used traditional method for controlling cereal aphids. Foliar application of broad-spectrum insecticides may reduce natural enemies’ population [10], contributing to pest resurgence. Imidacloprid, thiamethoxam and acetamiprid belong to neonicotinoid group of chemicals which act as systemic insecticides against sucking insect pests. The neonicotinoids have quick knockdown effect on target pests by interfering with transmission of impulse in the nervous system. The quick and excellent systemic and translaminar activity of these insecticides make them able to control sucking insect pests such as aphids, whiteflies and other virus transmission insects. Neonicotinoid applications reduce infection rate and spread of many crop viruses [11,12].

Heavy metals and pesticides are common examples of environmental toxicants, and they pose a major threat to the ecosystem’s function and structure. Heavy metals are naturally occurring elements with a high atomic weight and a density of more than 5 g/cm^3^. In comparison to their physical properties, heavy metals’ chemical properties are the most important in determining their environmental fate and toxicological behavior. Cadmium, lead, copper, and zinc all contribute to a frightening array of environmental and health issues [13].

In soil or plants, co-occurrence of toxicants can increase (synergism) or decrease (antagonism) joint toxicity; the joint toxicity can be stronger (synergistic), similar (additive), or weaker (antagonistic) than the single component [14]. Some factors controlling co-occurrence include bioavailability and biotransformation of these toxicants. Despite the high toxicity that can result from a single component, there is another complex interaction that can occur between two or more toxic mixtures, causing unpredictable toxicity [14]. For example, heavy metals can act as synergists by modulating detoxification enzymes (e.g., cytochrome P450 monooxygenases and glutathione S-transferases) in insects, thereby altering the internal concentrations of co-applied insecticides [15]. Conversely, antagonistic effects may arise when heavy metals compete with insecticides for binding sites or upregulate detoxification pathways [16,17].

These combined exposures are particularly relevant in agricultural ecosystems where both insecticides and heavy metals co-occur in soils and plant tissues. Although numerous studies have investigated the toxicity of neonicotinoid insecticides and heavy metals separately, there is a lack of studies evaluating these interactions under field conditions regarding the synergistic or antagonistic effects of insecticides and heavy metals combination against *R. padi*. The highest antagonism was found between Acetamiprid and soil Zn, Emamectin benzoate and soil Pb, Imidacloprid and Mn in soil and plant, Thiamethoxam and plant Zn. The highest synergistic relationships were found between Acetamiprid with Pb and Ni in plant, emamectin benzoate with Mn and Cd, in soil and plant’s Mn, imidacloprid and plant Zn, thiamethoxam and Pb in soil, and Cd in plant [14]. *R. padi aphids*, feeding on wheat plants, may simultaneously be exposed to insecticides and heavy metals through contaminated plants and agricultural practices, leading to complex toxic interactions that may enhance or suppress insecticidal activity. Therefore, understanding the interaction between neonicotinoid insecticides and heavy metals is essential for evaluating their combined toxicity against cereal aphids. The co-occurrence of pesticides and heavy metals in agricultural soils and wheat plant tissues may significantly affect the biological activity and toxicity of pesticides, leading to complex synergistic or antagonistic interactions in exposed invertebrates such as aphids [14,18,19]. Based on the above, the aim of this study was to determine the synergistic or antagonistic effect of four insecticides and five heavy metals on the median lethal concentrations (LC_50_ and LC_90_) and their toxic efficiency between 24 and 48 h post-exposure to the bird cherry–oat aphid.

## 2. Materials and Methods

The experiments were conducted at the Plant Protection Department, Faculty of Agriculture, Assiut University, Assiut, Egypt.

### 2.1. Insecticides Used

Insecticides were selected as the most commonly applied compounds by farmers to control wheat aphids in the Egyptian agro-ecosystem. The pesticides were obtained from the Central Agricultural Pesticide Laboratory (CAPL) in Dokki, Giza, Egypt. The four insecticides used were Emamectin benzoate (Avermectin, IRAC Group 6, Basha 1.9% EC), Imidacloprid (Neonicotinoid, IRAC Group 4A, Best 25% WP), Acetamiprid (Neonicotinoid, IRAC Group 4A, Mospildate 20% SP), and Thiamethoxam (Neonicotinoid, IRAC Group 4A, Actara 25% WG).

### 2.2. Insecticide Laboratory Bioassay Tests

The relative effectiveness of certain insecticide formulations of thiamethoxam, imidacloprid, acetamiprid, and emamectin benzoate were tested under laboratory conditions.

Insect: Field populations of the bird cherry–oat aphid, *R. padi*, were collected from naturally infested wheat fields under Assiut prevailing conditions during the 2023/2024 season. Infested wheat plants were carefully cut and transferred to the laboratory in plastic bags containing their host plant leaves. The collected aphids were reared under laboratory conditions for one generation on healthy wheat plants at a mean temperature of 28 ± 2 °C before being used in the toxicity bioassays. Aphids of nearly similar size and developmental stage were selected for all experiments to ensure uniformity of the tested population.

Laboratory bioassay: Toxicity of the investigated pesticides was assessed using the leaf-dip bioassay method [20]. Preliminary range-finding bioassays were conducted for each insecticide to identify concentration ranges producing approximately 10–90% mortality in *Rhopalosiphum padi*. Based on these preliminary tests, seven concentrations (1000, 100, 10, 1, 0.1, 0.001, 0.0001) were selected for each insecticide and used in the subsequent toxicity assays and Probit analysis. In addition to the commercial surfactant (Frotop^®^) at 40 mL/100 L, each insecticide was evaluated alone and in combination with one of the following heavy metals: Cr, Cu, Ni, Pb, and Cd. The tested heavy-metal concentrations were 0.1, 0.5, 0.2, 0.2, and 0.01 ppm for Cr, Cu, Ni, Pb, and Cd, respectively. These concentrations were selected to represent environmentally realistic, low-to-moderate contamination levels commonly encountered in agricultural ecosystems. All concentrations were below the FAO/WHO permissible limits for heavy metals in vegetable tissues, corresponding to approximately 5–66% of the respective permissible limits. Moreover, these concentrations were within, close to, or below the background ranges reported for plant tissues: Cr (0.006–18), Cu (5–30), Ni (0.1–5), Pb (0.1–10), and Cd (0.2–0.8 mg kg^−1^). Therefore, they were considered suitable for evaluating insecticide–heavy metal interactions under realistic agroecosystem exposure scenarios rather than under extreme contamination conditions [21,22]. Each concentration was evaluated using three independent replicates, with 10–15 aphid individuals of similar size per replicate, resulting in a total of 30–45 aphids tested per concentration. Aphids were dipped in the test solutions for 5 s and then allowed to air-dry for approximately 30 min at room temperature. Aphid control batches were similarly submerged in a mixture of water and the surfactant (Frotop^®^). After drying, aphids were individually transferred to 9-cm-diameter Petri dishes and maintained at 28 ± 2 °C, 60 ± 5% RH, and a 12:12 h (L:D) photoperiod. Aphid mortality was assessed 24 and 48 h after treatment using a binocular microscope. Aphids that were unable to move forward when gently stimulated were considered dead. Corrected mortality was calculated using Abbott’s formula [23]. Mortality data from all replicates were pooled, and Probit analysis was used to estimate LC_50_, LC_90_, 95% fiducial limits, and slope values.

### 2.3. Statistical Analysis

Data were subjected to the Probit analysis using the SPSS program version 28 to obtain the LC_50_, LC_90_, 95% fiducial limits (FL) and slope values for these pesticides according to Finney [24]. A significant level of mean separation (*p* < 0.05) was based on non-overlap between the 95% confidence intervals of LC_50_ values and expressed in ppm. Fold-change values were used as descriptive indicators of interaction strength and were not subjected to separate inferential statistical analysis.

## 3. Results

### 3.1. Test Insecticides Toxicity on R. padi After 24 h Exposure

**Figure 1:**
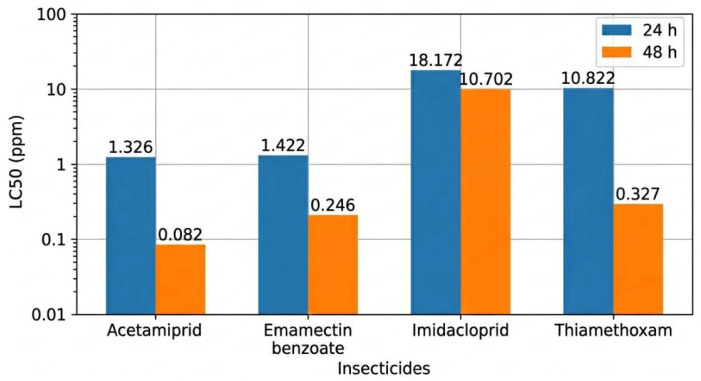
LC_50_ values (ppm) of four tested insecticides against *R. padi* at 24 h (blue) and 48 h (orange) of exposure (values are plotted on a logarithmic scale).

**Figure 2:**
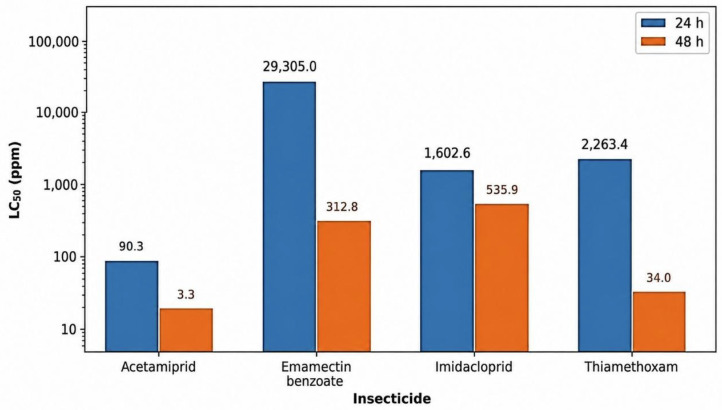
LC_90_ values (ppm) of four tested insecticides against *R. padi* (L.) at 24 h and 48 h of exposure (logarithmic scale).

Table 1 shows the LC_50_, LC_90_ and slope values that were calculated from the toxicity. These values corresponding to the four tested insecticides against *R. padi* after 24 h from treatment. Acetamiprid was the most toxic compound according to LC_50_ values, followed by emamectin benzoate, thiamethoxam, while imidacloprid was the least toxic one. Quantitatively, acetamiprid was more toxic than emamectin benzoate, thiamethoxam and imidacloprid by 1.07, 8.16, and 13.70-fold, respectively. According to LC_90_ values, the ranking order of toxicity of the tested compounds after 24 h from treatment without the heavy metal was acetamiprid > imidacloprid > thiamethoxam > emamectin benzoate, representing LC_90_ values 90.291, 1602.629, 2263.413, and 29,304.990 ppm, respectively. These results (Table 1) indicate that imidacloprid and emamectin benzoate exchanged their ranking position of toxicity in the same aphid colony. These results may be explained by the likely involvement of slope values to change the toxicity ranking (Table 1). The LC_50_ values at both 24 h and 48 h are illustrated comparatively in Figure 1. The LC_90_ values at both time points are similarly illustrated in Figure 2.

### 3.2. Effect of Certain Heavy Metals on the Toxicity of the Tested Insecticides Against R. padi After 24 h from Treatment

Table 1 shows the effect of adding heavy metals to the insecticides. According to the LC_50_ values after 24 h from treatment, the toxicity of thiamethoxam was increased with all tested heavy metals. LC_50_ values of thiamethoxam with Cr, Cu, Cd, Ni, and Pb were 0.59, 2.02, 0.15, 2.37, and 0.05 ppm, respectively. These results indicated that thiamethoxam was synergized by Cr, Cu, Cd, Ni, and Pb by 18.31, 5.36, 74.12, 4.58, and 225.46 fold, respectively, as compared with LC_50_ values of thiamethoxam alone. This finding indicated that Pb was the most effective heavy metal in synergizing thiamethoxam. With respect to imidacloprid, LC_50_ values in case of adding the heavy metals Cr, Cu, Cd, Ni, and Pb were 0.023, 25.79, 0.11, 1.20, and 1.60 ppm, respectively, as compared with LC_50_ values of imidacloprid alone which was 18.17 ppm. These results indicated that the toxicity of imidacloprid increased by adding each of the tested heavy metals except for Cu compared with LC_50_ of imidacloprid alone. The heavy metals Cr, Cd, Ni, and Pb synergized imidacloprid by 790.09, 160.81, 15.12, and 11.37 fold, respectively, showing that Cr was the most effective heavy metal in synergizing imidacloprid, followed by Cd, Ni, and Pb which was the least effective one. Cu was the only heavy metal that antagonized imidacloprid by decreasing its toxicity by 0.70-fold compared to imidacloprid alone (18.172 ppm).

### 3.3. Test Insecticides Toxicity Against R. padi After 48 h of Exposure

Table 2 shows LC_50_, LC_90_, and slope values of the insecticides with and without heavy metal 48 h after treatment. Values of LC_50_ for the insecticide alone was 0.082, 0.246, 10.702, and 0.327 ppm, for acetamiprid, emamectin benzoate, imidacloprid, and thiamethoxam, respectively. According to LC_50_ values 48 h after treatment, acetamiprid was more toxic than emamectin benzoate, thiamethoxam, and imidacloprid by 3.0, 3.99 and 130.51 folds, respectively. While depending on LC_90_ values, acetamiprid was also more toxic than the same corresponding insecticides by 94.23, 10.24, and 161 folds, respectively (Table 2). Comparing LC_50_ values of the four tested insecticides without heavy metals 48 h after treatment with that of 24 h after treatment, the toxicity of all tested insecticides was increased in the case of 48 h after treatment. Quantitatively, the toxicity increased by 16.17, 5.78, 1.70, and 33.10 folds for acetamiprid, emamectin benzoate, imidacloprid, and thiamethoxam, respectively (Table 1 and Table 2). Concerning LC_90_ values, the same trend was also found 48 h after treatment as compared with 24 h. Acetamiprid, emamectin benzoate, imidacloprid, and thiamethoxam were more toxic at 48 h compared to 24 h after treatment by 27.20, 93.70, 2.99, and 66.60 fold, respectively.

### 3.4. Effect of Certain Heavy Metals on the Toxicity of the Tested Insecticides After 48 h of Exposure

In case of adding Cr heavy metal to all tested insecticides (Table 2), LC_50_ values were 0.459, 0.930, 0.003, and 0.062 ppm, respectively. These results indicated that Cr heavy metal had decreased the toxicity of acetamiprid and emamectin benzoate by 0.18 and 0.26-fold, respectively. The same heavy metal increased the toxicity of imidacloprid and thiamethoxam by 3567.33 and 5.27-fold, respectively. Concerning Cu (Table 2), the LC_50_ value of emamectin benzoate insecticide alone was 0.246 ppm, while LC_50_ for the same insecticide plus Cu heavy metal was 0.207 indicating that Cu exerted a mild synergistic effect on emamectin benzoate (1.19-fold) 48 h after treatment. The LC_50_ values of acetamiprid, imidacloprid and thiamethoxam when adding Cu to each of them were 1.101, 13.041, and 0.445 ppm, respectively. These results indicated that Cu decreased the toxicity of these three insecticides by 0.07, 0.82, and 0.73-fold, respectively (antagonism).

Table 2 shows the LC_50_ and slope values of the four tested insecticides alone and mixed with Cd heavy metal. The results showed that LC_50_ value of each insecticide was decreased with Cd as compared with LC_50_ value of the insecticide alone. LC_50_ values for the insecticides with Cd were 0.001, 0.085, 0.032, and 0.066 ppm for acetamiprid, emamectin benzoate, imidacloprid, and thiamethoxam, respectively. These results indicated that adding Cd to the tested insecticides increased the toxicity of acetamiprid, emamectin benzoate, imidacloprid, and thiamethoxam by 82, 2.89, 334.44, and 4.95 fold, respectively.

### 3.5. Synergism and Antagonism of Heavy Metals on the Toxicity of the Tested Insecticides Against R. padi After 24 h and 48 h of Exposure

**Figure 3:**
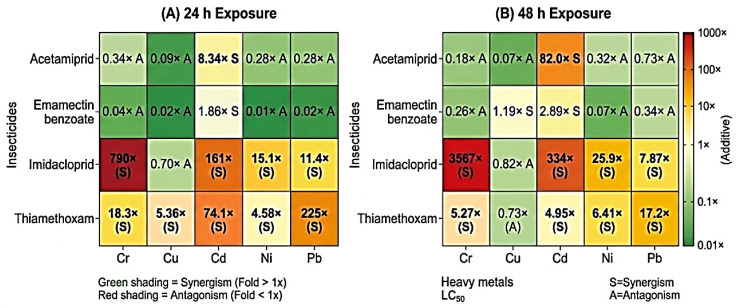
Heatmap of synergism/antagonism fold-change ratios for 20 insecticide-heavy metal combinations against *Rhopalosiphum padi* (L.) at (**A**) 24 h and (**B**) 48 h of exposure. Fold change = LC_50_ (insecticide alone)/LC_50_ (insecticide + heavy metal). Deep red/orange shading indicates synergism (fold > 1×); green shading indicates antagonism (fold < 1×). S = Synergism; A = Antagonism. Color intensity reflects magnitude of interaction.

To give a better idea about the direction and strength of the heavy metal and insecticide interactions, fold-change ratios (LC_50_ alone/LC_50_ + heavy metal) were determined for all combinations at both 24 h and 48 h (Table 3 and Table 4, respectively) and are graphically presented in Figure 3. Values above 1× correspond to synergism (heavy metal increases insecticide toxicity), values below 1× correspond to antagonism (heavy metal decreases toxicity).

After 24 h (Table 3), all the five heavy metals synergized thiamethoxam and the effect of Pb was the strongest (225.46×), followed by Cd (74.12×), Cr (18.31×), Cu (5.36×), and Ni (4.58×). For imidacloprid, Cr was the most effective synergist (790.09×) followed by Cd (160.81×), Ni (15.12×), and Pb (11.37×), while Cu showed antagonism (0.70×). Cd (8.34×) was the only heavy metal that synergized acetamiprid and all other heavy metals (Cr, Cu, Ni, and Pb) were antagonistic and reduced its toxicity by 0.09–0.34×. Similarly, the antagonistic effects of Cr, Cu, Ni, and Pb (0.006–0.04×) on emamectin benzoate and the mild synergism of Cd (1.86×) were observed.

The synergistic effects (Table 4) were generally more pronounced after 48 h than after 24 h and more so for imidacloprid-Cr (3567.33×) and imidacloprid-Cd (334.44×) than for 24 h, indicating a time-dependent increase in heavy metal–insecticide interactions. Acetamiprid-Cd was the most pronounced synergistic pair (82.00×) at 48 h while all other acetamiprid-heavy metal combinations were antagonistic. All heavy metals synergized thiamethoxam at 48 h except Cu (0.73× antagonism). Cu (1.19×) and Cd (2.89×) synergized emamectin benzoate, while Cr, Ni, and Pb were antagonistic. Overall, Cd was an effective synergist for all four insecticides at both time points confirming its role as the most universal synergist in this study.

**Figure 4:**
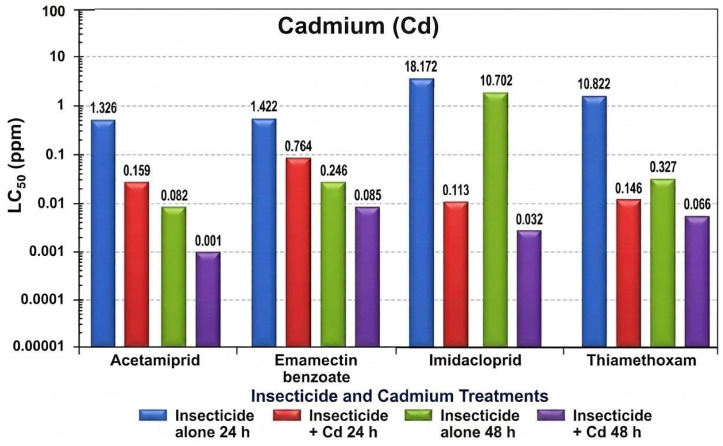
Effect of cadmium (Cd) on the LC_50_ (ppm) of four insecticides against *Rhopalosiphum padi* (L.) at 24 h and 48 h. Values are plotted on a logarithmic scale.

**Figure 5:**
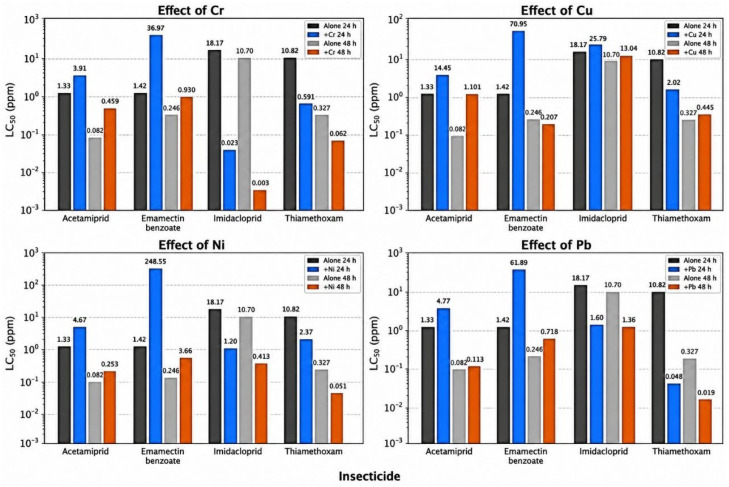
Effect of heavy metals Cr, Cu, Ni, and Pb on the LC_50_ (ppm) of four insecticides against *Rhopalosiphum padi* (L.) at 24 h and 48 h (logarithmic scale).

The Figure 4 shows that the addition of Cd in the four insecticides led to a significant decrease in LC_50_ values compared to that by each insecticide alone, revealing a stable synergism for two durations of exposure. At 24 h, the LC_50_ values for acetamiprid, emamectin benzoate, imidacloprid, and thiamethoxam decreased from 1.326, 1.422, 18.172, and 10.822 ppm alone to 0.159, 0.764, 0.113, and 0.146 ppm in the presence of Cd corresponding synergism ratios of 8.34-, 1.86-, 160.81-, and 74.12-fold, respectively. This enhancement effect was substantially potentiated at 48 h where the LC_50_ values further decreased to 0.001, 0.085, 0.032, and 0.066 ppm for the four insecticides in the same order with corresponding fold-increase of 82.00, 2.89, 334.44, and 4.95 individually. Collectively, these results identify Cd as a general potentiator that can increase the toxicity of the four insecticides at both the time points used in this study. The effects of the remaining heavy metals (Cr, Cu, Ni, and Pb) on insecticide LC_50_ values at both exposure times are illustrated in Figure 5.

## 4. Discussion

The present results (Table 1 and Table 2) demonstrated a clear increase in the toxicity of all tested insecticides against *R. padi* with increasing exposure time from 24 to 48 h. Based on LC_50_ values, acetamiprid showed the highest toxicity at 48 h (0.082 ppm), followed by emamectin benzoate, thiamethoxam, and imidacloprid, which was the least toxic. Similar trends were also observed for LC_90_ values, confirming the superior effectiveness of acetamiprid compared with the other compounds. Quantitatively, toxicity increased markedly to 48 h compared with 24 h, indicating a time-dependent enhancement in insecticidal performance. These findings agree with previous studies on aphid species, which reported that toxicity of neonicotinoids and their mixtures increase significantly with exposure time. For example, in *Aphis nerii*, the most toxic treatments after 48 h included acetamiprid-based mixtures and lambda-cyhalothrin + imidacloprid, and overall, LC_50_ values decreased with time, indicating higher toxicity at longer exposure periods. This supports the present results, where prolonged exposure enhanced insect susceptibility and reduced LC values [25]. Similar findings have been reported in previous studies on neonicotinoid insecticides, where toxicity increased with exposure duration and varied among compounds depending on their mode of action and binding affinity to nicotinic acetylcholine receptors [25,26]. Additionally, variability in susceptibility and differences in slope values have been linked to population heterogeneity in insect responses to insecticides [16]. These factors collectively explain the observed differences in LC values and toxicity ranking among the tested insecticides in the present study. It should be noted that the terms “synergistic” and “antagonistic” are used herein only to describe the direction of LC_50_ changes following heavy-metal co-exposure and should not be interpreted as formal Co-Toxicity Coefficient (CTC)-based interaction classifications. Because heavy metals were incorporated as fixed environmental co-stressors and were not evaluated individually through complete dose–response bioassays, classical CTC calculations were beyond the scope of the present study.

The present results demonstrated that the interaction between heavy metals and insecticides markedly influenced toxicity against *R. padi*, with both synergistic and antagonistic effects depending on the heavy metal type, insecticide, and exposure time. At 24 h, heavy metals, such as Pb and Cd, strongly synergized the toxicity of thiamethoxam and imidacloprid, while Cu showed an antagonistic effect in some cases. At 48 h, similar variability was observed, where Cr and Cd generally enhanced toxicity of several insecticides, whereas in some combinations Cr reduced the toxicity of acetamiprid and emamectin benzoate. These findings are consistent with previous studies reporting that heavy metals can significantly modify insecticide toxicity by affecting detoxification enzymes, oxidative stress pathways, and insect physiological responses. For example, toxic heavy metal exposure has been shown to interfere with cytochrome P450 monooxygenases and glutathione-S-transferases, leading to altered susceptibility to insecticides [27]. These results indicated that Cd consistently enhanced the toxicity of all tested insecticides under the conditions of the present study. One of the most common and sensitive soil toxicity indicators is the earthworm [17]. Wang et al. [28] studied the effect of combined toxicity of Cd with five types of insecticides on the earthworms. Their findings reported that 23 ternary mixtures showed different interactive effects. Among them, 11 mixtures exhibited synergistic effects, while five recorded antagonistic effects. These results revealed that synergistic interactions could occur more than antagonistic ones.

These results showed that some heavy metals enhanced insecticide toxicity, others reduced it, whereas some produced little or no measurable effect on insecticide toxicity. These findings are consistent with the concept that co-occurrence of toxicants in agroecosystems can produce complex and unpredictable joint effects depending on bioavailability and biotransformation pathways [18,29].

Several factors may influence the relationship between insecticides and heavy metals, including the chemical structure of the insecticide, method of application, the mode of resistance and chromosomal karyotype of the tested aphid which depend on the back history of pesticides used in the field infested with the tested aphid. Concerning slope values for all tested insecticides with or without heavy metals, most of those values were less than 1.0. This finding, as expected, indicated that the tested aphid colonies were relatively heterogenous. This result may be due to different colonies resulted from different females (parthenogenetic reproduction) and different karyotypes. Some of field-colonies of aphid probably with a normal chromosomal karyotype, some others with the common A1, 3—autosomal translocation and revertant individuals when the resistant aphids reverted from the resistant type to the susceptible one by the reversion of autosomal translocation to the normal karyotype may also be involved [30]. Extremely high LC_90_ values observed in some combinations may be attributed to shallow slope values and heterogeneity of aphid populations, resulting in wide confidence intervals and reduced precision of high-level mortality estimation.

Table 3 and Table 4 show the fold-change summary of the synergistic and antagonistic effects for all the insecticide-heavy metal interactions at 24 h and 48 h. These tables show that synergistic interactions were mostly observed (especially for thiamethoxam and imidacloprid), and antagonistic effects were predominant for emamectin benzoate and acetamiprid against more tested heavy metals. Imidacloprid + Cr at 48 h exhibited the highest level of synergism (3567.33×), followed by thiamethoxam + Pb at 24 h (225.46×), demonstrating the extreme sensitivities of such combinations. The differential responses—synergism for some combinations and antagonism for others—are likely driven by metal-specific interactions with insect detoxification enzyme systems; for example, cadmium and lead are known to inhibit CYP450 enzymes and GSTs, thereby potentiating insecticide toxicity, while copper may upregulate certain antioxidant and detoxification pathways, resulting in antagonism [15,19,27]. These results are in line with recent publications that heavy metals may greatly modify the toxicity of insecticides via interference with crucial detoxification mechanisms in insects [19].

The consistent toxicity-enhancing effect of Cd across all tested insecticides at both time points merits particular attention (Figure 3). Cadmium also strongly inhibits cytochrome P450 monooxygenases (CYPs) and glutathione S-transferases (GSTs), which are the main metabolic enzymes involved in neonicotinoids detoxification in insects [19]. Cd suppresses these detoxification enzymes so efficiently that it also blocks the breakdown metabolism of insecticides leading to higher internal concentrations and greater mortality. This process is well documented by earthworm research, in which Cd-insecticide compounds mostly synergized [28] and by more general indications that heavy metal co-exposure elicits oxidative stress responses in insects via production of reactive oxygen species (ROSs), which even debilitates superoxide dismutase (SOD) and catalase (CAT), the insect antioxidant defense mechanisms [17].

The marked synergism of Cr with imidacloprid and thiamethoxam, but antagonism with acetamiprid and emamectin benzoate, suggests that heavy metal–insecticide interactions are highly insecticide-class specific and depend on molecular structure, mode of action, and metabolic pathways of each compound. Chromium (Cr) at the tested concentration likely competes for binding sites on detoxification enzymes, with differential affinity depending on the chemical nature of each insecticide. Similarly, Pb consistently synergized thiamethoxam with one of the highest fold-change values observed (225.46× at 24 h and 17.21× at 48 h), supporting the hypothesis that Pb impairs neural signaling pathways in insects that overlap with the nicotinic acetylcholine receptor (nAChR) targets of neonicotinoids [31], thereby amplifying the neurotoxic effect. This hypothesis is proposed by the present authors based on the observed synergistic data; direct mechanistic evidence from electrophysiological or biochemical studies would be needed to confirm it.

Antagonistic effects, especially pronounced for emamectin benzoate with Cr, Cu, Ni, and Pb at 24 h, may reflect competitive inhibition at the target site level, or induction of efflux transporters and phase-II detoxification enzymes stimulated by sublethal heavy metal stress prior to insecticide exposure. These antagonistic outcomes are practically significant, as they imply that the efficacy of emamectin benzoate in heavy metal–contaminated field environments may be substantially reduced. This has implications for integrated pest management (IPM) strategies in agroecosystems where heavy metal contamination of soils and plant tissues is prevalent. The differential susceptibility of insecticides to heavy metal modulation may reflect the specific metabolic pathways targeted by each compound and the degree to which those pathways are disrupted by metal-induced enzyme inhibition or induction [19,32].

The higher synergistic effects at longer exposure times (24 h and 48 h), especially for imidacloprid + Cr (790.09× to 3567.33×) and acetamiprid + Cd (8.34× to 82.00×) suggest an ongoing accumulation of toxic burden with time. This phenomenon is in line with the time-enhanced toxicity of neonicotinoids reported in aphid species [25]. These temporal patterns imply that the detoxification enzyme systems of *R. padi* are increasingly overtaxed under continuous co-exposure, consistent with reports of cadmium-induced stress responses in aphids (*Megoura crassicauda*) to cadmium accumulation along food chains [33]. One limitation of the present study is that the interactions were evaluated under laboratory conditions only, without biochemical confirmation of detoxification enzyme activity or field validation under natural agroecosystem conditions. Therefore, further studies are needed to investigate the underlying physiological mechanisms and to validate these interactions under field conditions.

## 5. Conclusions

The present study revealed that the toxicity of four tested insecticides against *Rhopalosiphum padi* (L.) increased with increasing exposure time from 24 h to 48 h and acetamiprid exhibited the lowest LC_50_ values at both time intervals indicating that it was the most effective agent and imidacloprid was the least toxic. Among the heavy metals tested, only cadmium increased the toxicity of four insecticides at 24 and 48 h after treatment. Lead induced the strongest synergistic effect on thiamethoxam (225.46-fold at 24 h) and chromium was the best synergist to imidacloprid (3567.33-fold at 48 h). On the other hand, emamectin benzoate was antagonized by most of the tested heavy metals, especially at 24 h. The results of this study confirm that the presence of heavy metals in agricultural fields can greatly change the biological activity of insecticides, and this should be taken into consideration when planning pest management programs, especially in areas with high heavy metal contamination in soil or plant tissues.

## Figures and Tables

**Table 1:** Acute toxicity of the tested insecticides with and without heavy metals against *R. padi* after 24 h from treatment.

Treatment	Slope ± SE	LC_50_ (FL) 95%	LC_90_ (FL) 95%
Acetamiprid	0.699 ± 0.074	1.326 (0.840–1.964)	90.291 (44.667–251.251)
Emamectin benzoate	0.297 ± 0.046	1.422 (0.394–3.578)	29,304.990 (3826.733–1,135,214.083)
Imidacloprid	0.659 ± 0.085	18.172 (11.923–27.287)	1602.629 (616.697–7608.329)
Thiamethoxam	0.552 ± 0.058	10.822 (6.625–18.304)	2263.413 (771.395–11,020.887)
Acetamiprid + Cr	0.543 ± 0.066	3.906 (0.50–16.430)	894.923 (144.230–1,536,040.260)
Acetamiprid + Cu	0.666 ± 0.076	14.445 (9.155–21.606)	1213.721 (544.635–4067.247)
Acetamiprid + Cd	0.512 ± 0.062	0.159 (0.087–0.268)	50.902 (17.233–275.018)
Acetamiprid + Ni	0.263 ± 0.061	4.667 (1.688–15.645)	349,705.421 (8518.584–6,115,273,609.925)
Acetamiprid + Pb	0.288 ± 0.055	4.767 (1.822–12.522)	135,106.045 (7425.768–70,494,336.461)
Emamectin benzoate + Cr	0.779 ± 0.080	36.968 (25.899–52.839)	1633.590 (801.111–4587.004)
Emamectin benzoate + Cu	0.362 ± 0.055	70.952 (30.652–252.689)	245,105.434 (20,727.919–22,422,615.784)
Emamectin benzoate + Cd	0.446 ± 0.064	0.764 (0.338–1.407)	568.375 (153.552–5402.986)
Emamectin benzoate + Ni	0.563 ± 0.066	248.550 (150.017–468.522)	46,754.546 (13,085.006–342,001.702)
Emamectin benzoate + Pb	0.605 ± 0.081	61.889 (39.688–107.032)	8132.993 (2397.391–62,536.737)
Imidacloprid + Cr	0.431 ± 0.084	0.023 (0.006–0.048)	21.443 (4.895–518.829)
Imidacloprid + Cu	0.784 ± 0.072	25.785 (17.751–36.768)	1112.343 (595.132–2626.030)
Imidacloprid + Cd	1.174 ± 0.146	0.113 (0.065–0.164)	1.397 (1.021–2.184)
Imidacloprid + Ni	1.530 ± 0.165	1.202 (0.839–1.563)	8.273 (6.468–11.499)
Imidacloprid + Pb	0.615 ± 0.086	1.598 (0.807–2.587)	193.793 (81.794–823.647)
Thiamethoxam + Cr	1.136 ± 0.188	0.591 (0.260–0.925)	7.927 (5.637–14.159)
Thiamethoxam + Cu	0.872 ± 0.091	2.018 (1.118–3.125)	59.482 (38.758–105.606)
Thiamethoxam + Cd	0.923 ± 0.102	0.146 (0.078–0.228)	3.574 (2.398–6.122)
Thiamethoxam + Ni	0.283 ± 0.065	2.365 (0.815–6.251)	79,587.471 (2998.750–413,380,841.414)
Thiamethoxam + Pb	0.784 ± 0.105	0.048 (0.016–0.096)	2.066 (1.325–3.700)

LC_50_ = Lethal concentration required to kill 50% of the tested aphids, LC_90_ = Lethal concentration required to kill 90% of the tested aphids, FL = Fiducial limits at 95% confidence level; LC_50_ values were considered significantly different when 95% fiducial limits did not overlap.

**Table 2:** Acute toxicity of the tested insecticides with and without heavy metals against *R. padi* after 48 h from treatment.

Treatment	Slope ± SE	LC_50_ (FL) 95%	LC_90_ (FL) 95%
Acetamiprid	0.798 ± 0.088	0.082 (0.036–0.149)	3.319 (2.101–5.896)
Emamectin benzoate	0.413 ± 0.049	0.246 (0.073–0.584)	312.760 (115.781–1342.910)
Imidacloprid	0.754 ± 0.086	10.702 (7.054–15.314)	535.881 (267.999–1084.201)
Thiamethoxam	0.635 ± 0.062	0.327 (0.021–1.226)	33.990 (8.336–834.409)
Acetamiprid + Cr	0.572 ± 0.075	0.459 (0.116–1.102)	79.530 (42.826–187.049)
Acetamiprid + Cu	0.670 ± 0.081	1.101 (0.433–2.079)	90.305 (51.908–196.700)
Acetamiprid + Cd	0.460 ± 0.079	0.001 (0.000–0.003)	0.459 (0.218–1.311)
Acetamiprid + Ni	0.355 ± 0.063	0.253 (0.054–0.622)	1040.162 (194.646–28,978.533)
Acetamiprid + Pb	0.834 ± 0.086	0.113 (0.056–0.191)	3.882 (2.413–7.085)
Emamectin benzoate + Cr	0.602 ± 0.076	0.930 (0.309–1.925)	125.327 (67.733–303.511)
Emamectin benzoate + Cu	0.313 ± 0.053	0.207 (0.033–0.607)	2565.113 (447.180–69,528.741)
Emamectin benzoate + Cd	0.625 ± 0.078	0.085 (0.030–0.171)	9.488 (5.208–22.329)
Emamectin benzoate + Ni	0.636 ± 0.070	3.662 (1.711–6.425)	378.243 (202.127–910.352)
Emamectin benzoate + Pb	0.805 ± 0.092	0.718 (0.298–1.321)	28.089 (17.914–49.444)
Imidacloprid + Cr	0.708 ± 0.103	0.003 (0.001–0.008)	0.213 (0.131–0.409)
Imidacloprid + Cu	0.799 ± 0.072	13.041 (8.689–18.692)	524.117 (300.448–1108.600)
Imidacloprid + Cd	0.868 ± 0.153	0.032 (0.007–0.068)	0.950 (0.644–1.700)
Imidacloprid + Ni	1.634 ± 0.250	0.413 (0.190–0.648)	2.517 (1.932–3.368)
Imidacloprid + Pb	1.089 ± 0.109	1.360 (0.284–2.934)	20.422 (8.314–224.116)
Thiamethoxam + Cr	1.000 ± 0.288	0.062 (0.001–0.241)	1.182 (0.390–1.879)
Thiamethoxam + Cu	0.697 ± 0.096	0.445 (0.126–0.969)	30.669 (18.638–60.352)
Thiamethoxam + Cd	1.066 ± 0.123	0.066 (0.032–0.108)	1.044 (0.732–1.598)
Thiamethoxam + Ni	0.348 ± 0.069	0.051 (0.004–0.177)	243.644 (55.608–5575.646)
Thiamethoxam + Pb	0.858 ± 0.129	0.019 (0.004–0.046)	0.599 (0.368–0.996)

LC_50_ = Lethal concentration required to kill 50% of the tested aphids, LC_90_ = Lethal concentration required to kill 90% of the tested aphids, FL = Fiducial limits at 95% confidence level; LC_50_ values were considered significantly different when 95% fiducial limits did not overlap.

**Table 3:** Fold change indicating synergistic and antagonistic effects of insecticide–heavy metal combinations against *Rhopalosiphum padi* (L.) after 24 h of exposure.

Insecticide	Heavy Metal	LC_50_ Alone (ppm)	LC_50_ + Heavy Metal (ppm)	Fold Change	Interaction Type
Acetamiprid	Cr	1.326	3.906	0.34×	Antagonism
Acetamiprid	Cu	1.326	14.445	0.09×	Antagonism
Acetamiprid	Cd	1.326	0.159	8.34×	Synergism
Acetamiprid	Ni	1.326	4.667	0.28×	Antagonism
Acetamiprid	Pb	1.326	4.767	0.28×	Antagonism
Emamectin benzoate	Cr	1.422	36.968	0.04×	Antagonism
Emamectin benzoate	Cu	1.422	70.952	0.02×	Antagonism
Emamectin benzoate	Cd	1.422	0.764	1.86×	Synergism
Emamectin benzoate	Ni	1.422	248.550	0.006×	Antagonism
Emamectin benzoate	Pb	1.422	61.889	0.02×	Antagonism
Imidacloprid	Cr	18.172	0.023	790.09×	Synergism
Imidacloprid	Cu	18.172	25.785	0.70×	Antagonism
Imidacloprid	Cd	18.172	0.113	160.81×	Synergism
Imidacloprid	Ni	18.172	1.202	15.12×	Synergism
Imidacloprid	Pb	18.172	1.598	11.37×	Synergism
Thiamethoxam	Cr	10.822	0.591	18.31×	Synergism
Thiamethoxam	Cu	10.822	2.018	5.36×	Synergism
Thiamethoxam	Cd	10.822	0.146	74.12×	Synergism
Thiamethoxam	Ni	10.822	2.365	4.58×	Synergism
Thiamethoxam	Pb	10.822	0.048	225.46×	Synergism

Fold = LC_50_ (insecticide alone)/LC_50_ (insecticide + heavy metal). Fold > 1 = Synergism (heavy metal increases toxicity); Fold < 1 = Antagonism (heavy metal decreases toxicity). × = fold increase in toxicity over exposure time.

**Table 4:** Fold change indicating synergistic and antagonistic effects of insecticide–heavy metal combinations against *Rhopalosiphum padi* (L.) after 48 h of exposure.

Insecticide	Heavy Metal	LC_50_ Alone (ppm)	LC_50_ + Heavy Metal (ppm)	Fold Change	Interaction Type
Acetamiprid	Cr	0.082	0.459	0.18×	Antagonism
Acetamiprid	Cu	0.082	1.101	0.07×	Antagonism
Acetamiprid	Cd	0.082	0.001	82.00×	Synergism
Acetamiprid	Ni	0.082	0.253	0.32×	Antagonism
Acetamiprid	Pb	0.082	0.113	0.73×	Antagonism
Emamectin benzoate	Cr	0.246	0.930	0.26×	Antagonism
Emamectin benzoate	Cu	0.246	0.207	1.19×	Synergism
Emamectin benzoate	Cd	0.246	0.085	2.89×	Synergism
Emamectin benzoate	Ni	0.246	3.662	0.07×	Antagonism
Emamectin benzoate	Pb	0.246	0.718	0.34×	Antagonism
Imidacloprid	Cr	10.702	0.003	3567.33×	Synergism
Imidacloprid	Cu	10.702	13.041	0.82×	Antagonism
Imidacloprid	Cd	10.702	0.032	334.44×	Synergism
Imidacloprid	Ni	10.702	0.413	25.91×	Synergism
Imidacloprid	Pb	10.702	1.360	7.87×	Synergism
Thiamethoxam	Cr	0.327	0.062	5.27×	Synergism
Thiamethoxam	Cu	0.327	0.445	0.73×	Antagonism
Thiamethoxam	Cd	0.327	0.066	4.95×	Synergism
Thiamethoxam	Ni	0.327	0.051	6.41×	Synergism
Thiamethoxam	Pb	0.327	0.019	17.21×	Synergism

Fold = LC_50_ (insecticide alone)/LC_50_ (insecticide + heavy metal). Fold > 1 = Synergism (heavy metal increases toxicity); Fold < 1 = Antagonism (heavy metal decreases toxicity). × = fold increase in toxicity over exposure time.

## Data Availability

All the results obtained in this research are contained in the manuscript, and any questions can be directed to the corresponding authors.
